# Early experience with pulsed field ablation for atrial fibrillation in Central Asia: A case series

**DOI:** 10.1016/j.hroo.2025.06.015

**Published:** 2025-06-27

**Authors:** Ayan Abdrakhmanov, Zhansaya Yerkhanova, Serge Boveda, Zhanar Abdrakhmanova, Assel Chinybayeva, Bibigul Adilbekova, Zhanasyl Suleymen

**Affiliations:** 1 Heart Rhythm Scientific Research Institute, Astana Medical University, Astana, Kazakhstan; 2 Department of Cardiology, Medical Centre Hospital of the President’s Affairs Administration of the Republic of Kazakhstan, Astana, Kazakhstan; 3 Heart Rhythm Management Department, Clinique Pasteur, Toulouse, France

**Keywords:** Atrial fibrillation, Pulsed field ablation, Heart failure, Catheter ablation, Electroporation

**Key Findings**

▪ To our knowledge, this is the first reported case series of pulsed field ablation (PFA) for atrial fibrillation in Central Asia.
▪ All patients remained in sinus rhythm at 3- and 6-month follow-up.
▪ In patients with heart failure with mildly reduced ejection fraction, PFA led to improved left ventricular function and reduced left atrial volume index.
▪ No atrial fibrillation recurrence or significant arrhythmias occurred during follow-up.

Pulsed field ablation (PFA) is a novel, nonthermal ablation modality increasingly used for the treatment of atrial fibrillation (AF)^1^, with a favorable safety profile and promising efficacy demonstrated in large multicenter studies.^2^, ^3^, ^4^ However, data from resource-constrained regions, particularly Central Asia, remain scarce.

We report preliminary outcomes from 14 consecutive patients who underwent PFA for symptomatic, drug-refractory paroxysmal or persistent AF at our institution between January 2024 and January 2025. The FARAPULSE PFA system (Boston Scientific, Marlborough, MA) was used in all procedures. Pulmonary vein isolation was successfully achieved in 100% of cases without major procedural complications, including stroke, esophageal injury, phrenic nerve palsy, or vascular events.

**Figure 1:**
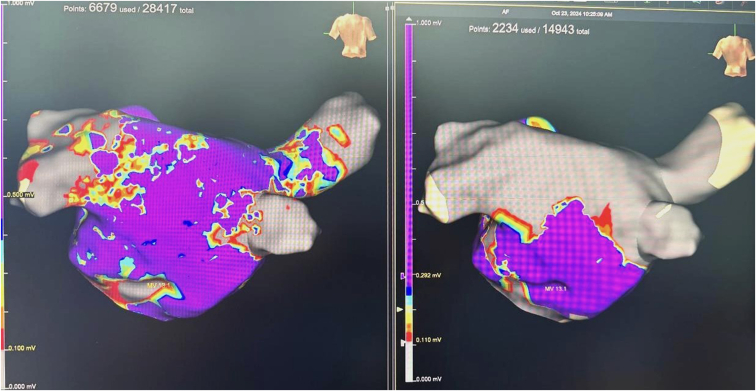
Preablation and postablation 3-dimensional electroanatomic mapping posterior wall ablation after multiple PVIs using PFA in persistent AF. AF = atrial fibrillation; PFA = pulsed field ablation; PVI = pulmonary vein isolation.

Additionally, for patients with persistent and long-standing persistent AF, supplemental linear ablation using the FARAWAVE catheter was performed in the roof and posterior wall of the left atrium (Figure 1).

At 3- and 6-month follow-up, all patients remained in sinus rhythm as confirmed by Holter monitoring and clinical assessment. In a subgroup of patients with heart failure with mildly reduced ejection fraction (n = 4), transthoracic echocardiography demonstrated a mean improvement in left ventricular ejection fraction from 44.8% to 52.8%. In addition, the mean left atrial volume index decreased from 45.8 mL/m^2^ to 42.2 mL/m^2^ after PFA (Table 1). No patients experienced AF recurrence or significant arrhythmias during the follow-up period.

**Table 1:** Echocardiographic outcomes in patients with HFmrEF after PFA

Patient	LA volume index before PFA (mL/m^2^)	LA volume index after PFA (mL/m^2^)	EF before PFA (%)	EF after PFA (%)
1	34.4	34.2	48	62
2	63.0	60.0	45	48
3	45.7	45.0	42	48
4	40.1	29.4	44	53

EF = ejection fraction; HFmrEF = heart failure with mildly reduced ejection fraction; LA = left atrial; PFA = pulsed field ablation.

Our experience aligns with the safety and efficacy outcomes reported in recent large studies, such as the MANIFEST-17K registry^2^ and the ADVENT trial^3^, which demonstrated high procedural success rates and low complication rates with PFA. Importantly, our findings suggest that PFA can be safely and effectively implemented in health-resource-constrained environments, providing a valuable alternative for patients with complex arrhythmia profiles, including those with structural heart disease and previous ablation failures.

In conclusion, PFA seems to be a feasible, safe, and effective ablation strategy for AF in Central Asia, including in patients with comorbid heart failure and ablation history.
